# Molecular purging of multiple myeloma cells by *ex-vivo *culture and retroviral transduction of mobilized-blood CD34^+ ^cells

**DOI:** 10.1186/1479-5876-5-35

**Published:** 2007-07-12

**Authors:** Sara Deola, Samantha Scaramuzza, Roberto Sciarretta Birolo, Massimiliano Cergnul, Francesca Ficara, Jonathan Dando, Claudia Voena, Sergio Vai, Marta Monari, Enrico Pogliani, Gianmarco Corneo, Jacopo Peccatori, Silvia Selleri, Claudio Bordignon, Maria Grazia Roncarolo, Alessandro Aiuti, Marco Bregni

**Affiliations:** 1 San Raffaele Telethon Institute for Gene Therapy (HSR-TIGET), Scientific Institute H.S. Raffaele, Milan, Italy; 2 Hematology and BMT Unit, Scientific Institute H.S. Raffaele, Milan, Italy; 3 Hematology and Bone Marrow Transplant Unit, Hospital S. Gerardo, Monza, Italy; 4 Department of Transfusion Medicine, Clinical Center, National Institutes of Health, Bethesda, MD 20892, USA; 5 Department of Biomedical Sciences and Human Oncology and Center for Experimental Research and Clinical Studies, University of Turin, Turin, Italy; 6 Department of Human Morphology, University of Milan, Milan, Italy

## Abstract

**Background:**

Tumor cell contamination of the apheresis in multiple myeloma is likely to affect disease-free and overall survival after autografting.

**Objective:**

To purge myeloma aphereses from tumor contaminants with a novel culture-based purging method.

**Methods:**

We cultured myeloma-positive CD34^+ ^PB samples in conditions that retained multipotency of hematopoietic stem cells, but were unfavourable to survival of plasma cells. Moreover, we exploited the resistance of myeloma plasma cells to retroviral transduction by targeting the hematopoietic CD34^+ ^cell population with a retroviral vector carrying a selectable marker (the truncated form of the human receptor for nerve growth factor, ΔNGFR). We performed therefore a further myeloma purging step by selecting the transduced cells at the end of the culture.

**Results:**

Overall recovery of CD34^+ ^cells after culture was 128.5%; ΔNGFR transduction rate was 28.8% for CD34^+ ^cells and 0% for CD138-selected primary myeloma cells, respectively. Recovery of CD34^+ ^cells after ΔNGFR selection was 22.3%. By patient-specific Ig-gene rearrangements, we assessed a decrease of 0.7–1.4 logs in tumor load after the CD34^+ ^cell selection, and up to 2.3 logs after culture and ΔNGFR selection.

**Conclusion:**

We conclude that *ex-vivo *culture and retroviral-mediated transduction of myeloma leukaphereses provide an efficient tumor cell purging.

## Background

The standard therapy for multiple myeloma is high-dose chemotherapy with autologous reinfusion of hematopoietic stem cells [1-3]. Molecular evidences indicate that residual cancer cells contaminate the transplant, and several reports estimate a tumor burden of 10^4 ^– 10^9 ^plasma cells per transplant [4,5]. Although their role in determining patients' overall survival is still unclear, a correlation has been shown between a plasma cell contamination >2 × 10^5^/lt and an early relapse [6,7]. In an effort to overcome this problem, a purging approach of aphereses was developed with a positive selection of CD34^+ ^cells, but no significant clinical advantage was achieved with this method [8]. Recent studies show that 75% of aphereses are still contaminated with plasma cells after the CD34^+ ^selection [9]. Other purging approaches exploited a more restricted phenotypic selection to eliminate tumor contaminants, such as CD34^+^/lin^-^/Thy1^+ ^[10,11], or combined selections (CD34^+^/CD19^-^) [12]. For both methods, preliminary clinical results indicate good purging achievements, but high infection rates, and poor bone marrow reconstitution results, due to the effects of progenitor- and T-cells depletion [11]. Barbui et al. described a purging approach based on negative selection of mobilized blood stem cells [13], in which they achieve safe engraftment results with purged cell grafts, but without advantages in the overall survival of patients. A wide variability of purging results – ranging from 0 to 7 logs depletions – is achieved by these phenotype-based purging methods. Still no clinical trial so far established a defined threshold of purging necessary to reduce the risk of relapse while maintaining a safe clinical feasibility.

We investigated the efficiency of a culture-based purging of myeloma aphereses.

Myeloma plasma cells exhibit a high mortality in culture (50–95% in 9 days) [14]; we exploited this biological behaviour to perform a culture-based purging of myeloma CD34-selected leukaphereses. We designed a 4-day cytokine-culture procedure, adequate for a safe maintenance of the CD34^+ ^cell multilineage phenotype, but unfavourable to plasma cells. Moreover myeloma cells display a very low retroviral-mediated transduction rate, even after repeated infection cycles (1.5–5.4%) [14,15], therefore we structured the protocol to allow the insertion and the expression of retroviral genes in the CD34^+ ^cells. Through this approach, we directed the selectable marker ΔNGFR to the CD34^+ ^cell population, adding a further purging possibility. ΔNGFR has already been utilized in T cell populations to control GvHD in allogeneic bone marrow transplants [16,17]. To assess the effectiveness of purging, we amplified patient-specific, clonal tumor immunoglobulin heavy-chain (IgH) rearrangements before and after CD34^+ ^cell culture procedure and retroviral transduction.

We present here the results of culture and transduction of primary cells from leukaphereses of 19 multiple myeloma (MM) patients, undergoing high-dose chemotherapy and peripheral blood cell autografting.

## Methods

### Cell processing

Mobilized peripheral blood (MPB) and bone marrow (BM) cells were collected from MM patients enrolled in high-dose chemotherapy programs, approved by IRB at HSR, after informed consent. Mononuclear cells were purified by density centrifugation using the lymphocyte separation medium Lymphoprep (Nycomed Pharma, Asker, Norway).

CD34^+ ^cells were positively selected using the following immunomagnetic separation devices: mini-, and midi-MACS (CD34^+ ^MultiSort Kit, Miltenyi Biotec, Bergisch Gladbach, Germany), cliniMACS (Miltenyi Biotec), ISOLEX300i (Baxter Healthcare, Irvine, CA, USA), according to the manufacturer's instructions. CD138^+ ^cells were selected with mini-MACS device, using direct conjugated anti CD138 microbeads (Miltenyi Biotec). Immunoselected cells were analyzed by flow-cytometry, to determine the purity of selections.

### Retroviral supernatant production

BML-1 Moloney Murine Leukemia Virus (MoMLV) based retrovirus, containing the ΔNGFR marker gene under the control of LTR promoter [18] was collected from producer cells, kindly provided by Roche Diagnostics GmbH. Producer cells were expanded in 200 μl/cm^2 ^Iscove's modified Dulbecco's medium (IMDM, Bio Whittaker, Verviers, Belgium) supplemented with 10% fetal bovine serum (FCS, Euroclone, Wetherby, West Yorkshire, UK), 100 U/ml penicillin-streptomycin, and 2 mM L-glutamine at 5 × 10^4 ^cells/cm^2^, at 37°C with 5% CO_2_. The medium was replaced after 48 hours, 72 hours and 84 hours reducing the volume to 66 μl/cm^2 ^and the temperature to 33°C [19]. Viral supernatant (SN) was collected with 12-hours serial collections, 0.45 filtered and -80°C frozen before use.

### Culture, transduction and selection of transduced cells

CD34^+ ^cells were cultured in X-Vivo 10 (Bio Whittaker) serum free medium containing the cytokines thrombopoietin (PeproTech, Rocky Hill, NJ, USA), FLT3-Iigand (PeproTech), stem cell factor (R&D Systems Minneapolis, MN, USA) at 50 ng/ml (thereafter designated complete medium) at 5–6 × 10^5^cells/cm^2 ^for 84 hours. Transduction of CD34^+ ^cells were performed in complete medium, at the same cell concentration. Cells were prestimulated for 24 hours, and overnight transduced with the BML-1 retroviral vector on RetroNectin (TaKara Biomedicals, Kyoto, Japan)-coated non-tissue culture-treated plates, or T75 flasks, with 4:1 v/v SN/culture medium, supplemented with 5× concentrated cytokines. Fresh complete medium was replaced after virus withdrawal and cells were cultured for further 48 hours. At the end of the culture, cells were collected, washed, and incubated with anti-NGFR biotinylated antibody at 2.5 × 10^6 ^cell/ml for 30 minutes at 4°C and then with 10 μl streptavidin (SA)-conjugated microbeads (Miltenyi Biotec) for 15 minutes at 4°C. ΔNGFR^+ ^cells were then isolated by mini-MACS immunoselection device. ΔNGFR positive and negative fractions were analyzed by flow-cytometry for CD34, CD45, CD138, and ΔNGFR antigens. Clonal transduction of CD138^+ ^cells was performed in U-bottom 96-well plates with 4:1 SN/culture medium only in wells scored positive for 1 cell at light microscope. Prestimulation was performed with 40 μl of complete medium, and after 24 hours, 128 μl of virus, complemented with 32 μl of 5× cytokines, were directly added to each well. To avoid disturbance of the cells, transduction was stopped by substituting 150 μl of medium with fresh complete medium. Doubling of single cells was scored daily by microscopy and viability of cells using Trypan blue exclusion assessed at the end of transduction.

### Immunofluorescence staining and flow cytometric analyses

Cells were stained with conjugated monoclonal antibodies (mAbs) in 100 μl PBS, 0,1% sodium-azide, 0,3% BSA (PBS FACS) at 4°C for 25 minutes, after the staining cells were washed and resuspended in PBS FACS. For biotinylated mAbs a secondary staining with streptavidin-PE/FITC was performed. The following mAbs were used: CD34-PE (Becton Dickinson, San Josè, California, USA), CD45-FITC/TC (Caltag, Burlingame, California, USA), CD138-FITC (Valter Occhiena, Torino, Italy), CD38-TC (Caltag), SA-FITC/PE/TC (Caltag), biotinylated-NGFR. Isotype-identical mAbs IgG1-FITC/PE/TC and biotinylated-IgG1 (Caltag) served as control. Samples were acquired with FacsScan device (Becton Dickinson). Data were analyzed using CellQuest software (Becton Dickinson).

### *In vitro *clonogenic cell assays

Long-term marrow cultures (LTMC) were performed according to a described procedure [20]. Briefly, 70000 CD34^+^/ΔNGFR^+ ^cells were seeded on top of MS5 murine stromal cells in Myelocult medium (StemCell Technologies, Vancouver, BC, CA), added with 10 ng/ml IL6 (Pepro Tech) and half of the medium was weekly replaced. After three weeks of culture, cells were resuspended in Methocult GF medium (StemCell Technologies) and plated in duplicate in a (Colony Forming Units-Cells) CFU-C assay. Colonies were scored two weeks later, individually picked and DNA was extracted for PCR analysis. DNA extraction was performed adding, for each colony, 25 μl of KCl lysis buffer and protease K at 50 μg/ml. Colonies were incubated overnight at 37°C and heat inactivated for 15' at 94°C. KCl lysis buffer consists of 1:1 v/v mix of solution A (100 mM KCl, 10 mM Tris pH 8.3, 2.5 mM MgCl_2_) and solution B (10 mM Tris pH 8.3, 2.5 mM MgCl_2_, 1% Tween-20, 1% NP-40). DNA was then analyzed for the presence of ΔNGFR transgene, and patient-specific myeloma markers.

### PCR assays

To determine the presence of ΔNGFR transgene, DNA was amplified with primers 5'-LΔ1: GGCCGTTGGATTACACGGTC and 3'-MAGO: CCTACAGGTGGGGTCTTTCA. PCR reactions were carried out in a final volume of 25 μl, with 1 μM primers, 2.5 mM MgCl_2_, 0.25 mM dNTPs and 1.25 U Taq Gold (Perkin Elmer, Wellesley, MA, USA), at following conditions: 7 minutes at 94°C, 50 seconds at 94°C, 50 seconds at 60°C and 50 seconds at 72°C for 40 cycles, 5 minutes at 72°C. As a control for the presence of DNA, HLA genes were amplified (primer 5':GTGCTGCAGGTGTAAACTTGTACCAG and primer 3':CACGGATCCGGTAGCAGCGGTAGAGTTG). PCR conditions were the same as above, except for annealing temperature that was 56°C.

To detect tumor cells, the clonal variable region (VDJ) rearrangements of the IgH genes of each patient were amplified using 5' consensus primers derived from the conserved sequences of the variable region and 3' consensus primer derived from the joining region as previously described [21]. PCR products were sequenced and sequences from CDR2 and CDR3 regions were used to design patient-specific tumor primers. Oligonucleotides were subsequently tested for specificity using polyclonal DNA from normal individuals as negative controls. For each patient, CD34^+ ^cells before and after culture, and after ΔNGFR immunoselection were amplified to detect residual myeloma cells. Cells were directly resuspended at 1 × 10^5^/50 μl in KCl lysis solution for genomic DNA extraction (as described above) and PCR amplifications were performed using 5, or 10 μl of DNA. A first round PCR was performed using patient specific VH family primers and a second round was performed amplifying 1 μl of the first PCR product with patient-specific primers. The sensitivity was 10^-4^-10^-5 ^[21].

Limiting dilution assays were performed serially diluting DNA with water, in 0.5 log increments and each dilution was subsequently amplified with patient-specific primers. At least five PCR reactions were performed for each dilution level from the level in which reactions were positive up to the level in which all reactions were negative for clonal markers. Polyclonal DNA was always included as negative control. This technique has been previously validated by diluting a known number of tumor cells in normal marrow or peripheral blood cells. An indirect quantification of the tumor load in the positive samples of the dilution was performed according to the statistical methods of likelihood maximization and χ^2 ^minimization (MC), derived from the single-hit Poisson model, as described by Taswell [22]. Combined frequency estimates were obtained by analyzing with MC estimator the pooled frequency determinations. Comparison of frequencies between groups was performed through evaluation of confidence interval (CI) and probability value, and CI for the ratio of 2 frequencies. All calculations were performed using MATLAB (version 5, The Math Works Inc.).

## Results

### Culture of myeloma aphereses, transduction and selection procedures

**Figure 1:**
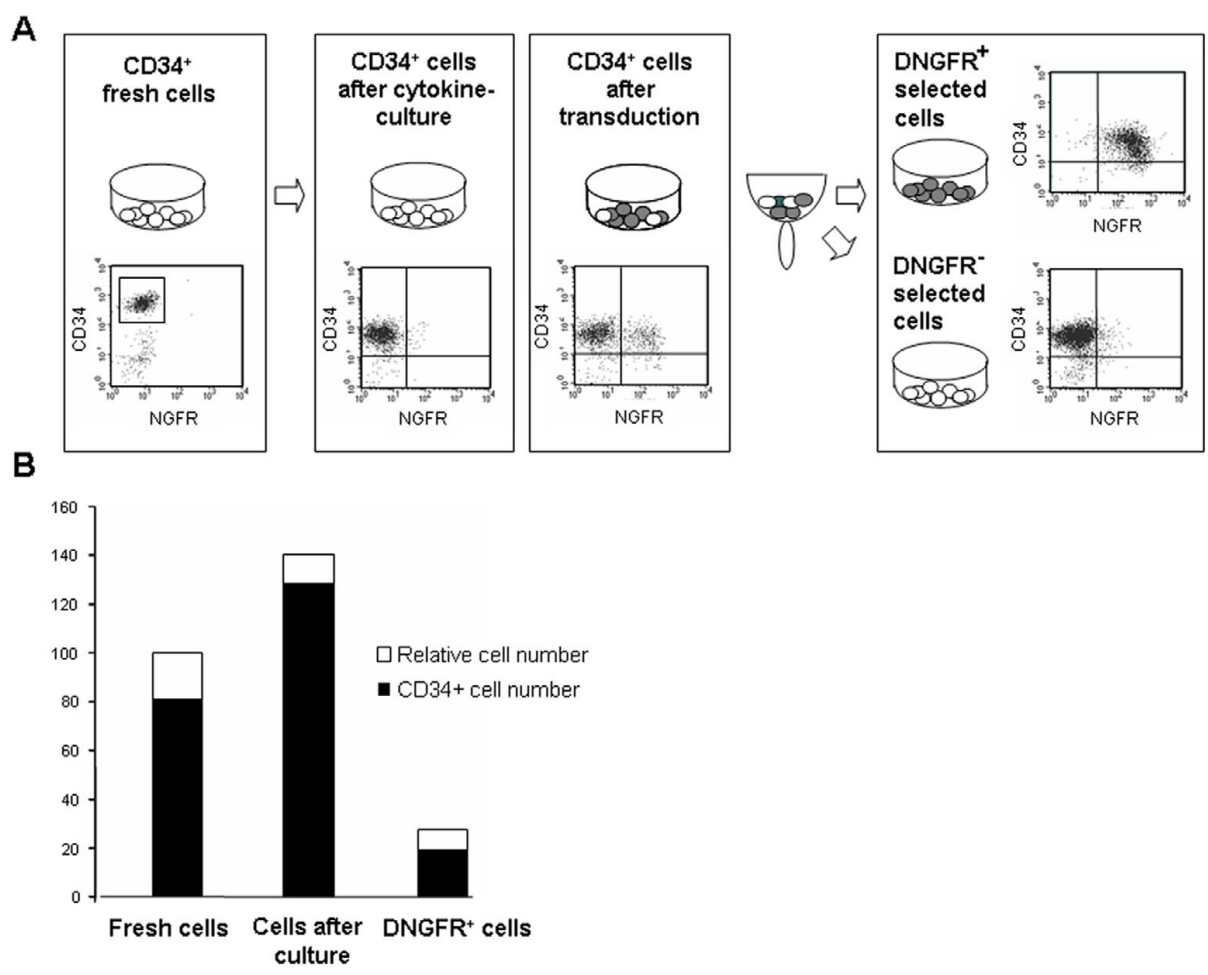
**MPB CD34^+ ^cells of myeloma patients before and after culture, and transduction**. (A) Phenotypic analyses of CD34+ cells before and after cytokine culture, ΔNGFR transduction, and selection of transduced cells. (B) Relative fold expansion in culture, and recovery after transduction and selection of total cells (open bars) and CD34+ cells (black bars), were measured, in comparison with the initial cell population (fresh cells).

Nineteen MPB leukaphereses products were CD34-selected by miniMACS (n = 15), midiMACS (n = 1), cliniMACS (n = 1) or ISOLEX immunoselection system (n = 2). Mean purity of CD34^+ ^cells was 80.6 ± 19% (Table 1). CD34^+ ^cells were cultured for 84 hours, in complete medium. We previously described that this protocol allows a safe expansion of multipotent progenitors, without impairing their reconstitution capacity in severe combined immunodeficiency-humanized (SCID-hu) mice [18]. A portion of the CD34^+ ^cells was exposed to a retroviral transduction: after 24 hours of culture with complete medium, cells were overnight transduced with a MoMLV-derived retroviral vector, carrying the ΔNGFR gene [17]. After transduction, viral supernatant was replaced with fresh complete medium, and cells were allowed to express the transgene for further 48 hours (total culture time 84 hours, as in the non-retroviral exposed cytokine-culture). Transduced cells were immunoselected by ΔNGFR expression with MACS immunoselection system (Figure 1A), as previously described [23]. Mean CD34^+ ^cells transduction rate was 28.9 ± 12%, and purity of ΔNGFR immunoselected CD34^+ ^cells was 92.5 ± 5%.

**Table 1:** Results of CD34^+ ^cell selection, culture and transduction

**Patient**	**Number of CD34^+ ^selected cells (× 10^3^)**	**Purity of CD34^+ ^selected cells (%)**	**Transduction (%)**	**Number of ΔLNGFR selected cells (× 10^3^)**	**Purity of ΔLNGFR selected cells (%)**	**%CD34^+ ^cells among ΔLNGFR selected cells**	**CD34^+ ^transduced cells vs initial CD34^+ ^cells (ratio)**
MM 1	1082.0	78.6	29.8	156.2	92.8	72.4	13.3
MM 2	1913.0	33.6	20.9	36.0	85.5	43.6	24.4
MM 3	887.0	87.3	32.4	456.0	93.2	60.2	35.4
MM 4	1239.0	91.2	26.7	394.6	96.9	45.1	15.8
MM 5	1229.0	95.8	20.1	238.6	85.4	62.4	12.7
MM 6	1576.0	80.6	32.1	1520.0	94.5	62.2	74.4
MM 7	4285.0	37.1	31.4	729.0	90.4	67.6	31.0
MM 8	341.0	96.8	8.5	96.4	91.7	79.7	23.3
MM 9	1000.0	86.1	38.5	490.0	89.3	68.5	39.0
MM 10*	1000.0	97.2	56.1	266.0	89.3	78.2	21.4
MM 11	500.0	77.6	33.7	78.0	77.9	75.0	15.1
MM 12	15000.0	97.2	17.0	3900.0	88.0	76.3	20.4
MM 13	1000.0	50.4	26.0	175.0	95.0	71.0	24.7
MM 14*	22300.0	98.0	56.2	7990.0	97.8	67.5	24.1
MM 15	4400.0	76.0	18.0	576.0	93.3	79.0	13.8
MM 16	2890.0	79.0	20.0	396.0	97.3	84.0	14.6
MM 17	900.0	95.0	28.5	148.0	97.4	66.0	11.4
MM 18	1740.0	78.4	26.0	234.0	95.4	38.4	6.6
MM 19	2890.0	78.0	30.0	560.0	99.8	67.9	16.9
MM 20	2100.0	98.7	26.5	170.0	98.8	89.8	7.4
*Mean ± SD*		*80.6 ± 19%*	*28.9 ± 12%*		*92.5 ± 5%*	*67.7 ± 13%*	*22.3 ± 15%*

CD34^+ ^enriched cells of 19 patients were transduced with the ΔLNGFR retroviral vector, selected for transgene after 48 hours, and analyzed by flow cytometry. The last column shows the ratio between the number of CD34^+ ^cells in the ΔNGFR^+ ^selected cell population, and the initial number of CD34^+ ^cells. * cells derived from the same patient.

At the end of the procedure, 67.7 ± 13% of ΔNGFR selected cells still retained the CD34 phenotype, indicating a recovery of 22.3 ± 15% of the initial CD34^+ ^cell number (Table 1).

We did not observe differences in results when we performed the procedure with high numbers of CD34^+ ^cells (Table 1: exp. MM12, and MM14).

Mean fold-expansion of cytokine-cultured cells was 1.4, and CD34+ cells after culture were 128.5% of the initial number. CD34^+ ^antigen expression was 91.8 ± 4% after cytokine culture, and 67,7 ± 13% after transduction and selection of ΔLNGFR^+ ^cells. Recovery of total cells and CD34^+ ^cells in a representative experiment is shown in Figure 1B.

### Molecular purging

To assess the results of myeloma-cell purging, we developed patient-specific primers for tumor-specific monoclonal immunoglobulin gene rearrangements in 9 patients (MM#3,6,10/14,12,13,15,16,18 and 19). We amplified by PCR complementary determining regions I and III of the heavy chain VDJ immunoglobulin fragments, and we monitored monoclonal residual rearrangements in the apheresis product, after CD34^+ ^selection, after culture and after transduction. Mononuclear cells, CD34^+ ^fresh cells, cytokines-cultured cells, and ΔLNGFR-positive and -negative selected fractions were analyzed in 10 experiments, and DNA was amplified for myeloma specific clonal rearrangements.

Undiluted DNA was first amplified in 5 PCR reactions for each patient. Only in samples where sufficient material was available (n = 6), serial 0.5 log dilutions of DNA were performed before amplification, to allow a quantification of the purging. Results of the amplifications were interpreted with a Poisson estimate [22]. Since no quantitative real-time PCR has been performed, the results should be taken in relative terms (logdecreaseoftheinitialcell frequency). When we amplified DNA from mobilized aphereses, 83.3% of samples showed detectable myeloma contaminants, and 77.8% of the samples still retained myeloma cells after CD34^+ ^antigen selection (Table 2, gray shaded cells). The amount of tumor decrease after this first purging-step ranged from <0.67 to 1.36 logs. After *ex-vivo *cytokine culture, 57.1% (4 out of 7) tumor-positive CD34^+ ^samples lost myeloma specific rearrangements (MM6,14,18,19), with an estimation of 0 to >2.35 logs purging, with respect to the initial tumor load. We then measured how cell exposure to retroviral vector, and selection of transgene-expressing cells contributed to the purging. All the samples purged after cytokines-culture were also PCR negative for myeloma specific rearrangements after transduction and selection of the ΔNGFR^+ ^cell fraction. Among the 3 ΔNGFR^+ ^selected samples still positive for myeloma specific rearrangements, we observed a further decrease of myeloma contamination in samples MM15 and MM16 (range of purging: 0–5 to 1 log). Comparing the frequencies of tumor load in the different pooled data, we found a strong statistical significance between fresh CD34^+ ^cells vs. CD34^+ ^cells after culture, and after ΔNGFR selection (Table 3). When we compared the purging results of the culture with the ΔNGFR^+ ^cell fraction, we failed to find a relevant statistical significance (p > 0.1). This result suggests that the culture itself plays a major role in the purging.

**Table 2:** Results of tumor cell purging in mobilized blood cells before and after serial purging steps

**Tumor cell frequency**				
**Log decrease**				

**Exp**.	**Mobilized blood aphereses**	**CD34+ selected fresh cells**	**CD34+ cells after culture**	**NGFR+ selected cells**

**MM 3**	< 1:49334	< 1:49334	N.D.	< 1:49334
	**0**	**0**	**-**	**0**
**MM 6^#^**	1:2449	1:11791	< 1:89606	< 1:89606
	**0**	**0.68**	**> 1.56**	**> 1.56**
**MM 10**	> 1:6211	> 1:28818	N.D.	1:28852
	**0**	**< 0.67**	**-**	**0.67**
**MM 12^#^**	1:4239	< 1:89606	< 1:89606	< 1:89606
	**0**	**> 1.32**	**> 1.32**	**> 1.32**
**MM 13^#^**	1:10646	< 1:89606	< 1:89606	< 1:89606
	**0**	**> 0.92**	**> 0.92**	**> 0.92**
**MM 14^#^**	1:490	1:11162	< 1:109697	< 1:109697
	**0**	**1.36**	**> 2.35**	**> 2.35**
**MM 15^#^**	N.D.	1:12789	1:2669	1:26667
	N.D.	**0**	**0**	**1**
**MM 16**	N.D.	> 1:12422	1:28852	1:89256
	N.D.	**0**	**0.37**	**0.86**
**MM 18^#^**	N.D.	1:238	< 1:44823	< 1:44823
	N.D.	**0**	**> 2.27**	**> 2.27**
**MM 19**	N.D.	1:89286	< 1:89606	< 1:89606
	N.D.	**0**	**> 0**	**> 0**
**POOLED DATA**	1:5291	1:12270	1:84602	1:208333
	**0**	**0.36**	**1.2**	**1.59**

Mobilized blood cells were analyzed after different purging steps for tumor load, where the specific MM marker was available. DNA from 1 × 10^4^, or 2 × 10^4 ^cells was amplified to detect MM contaminants. For 6 patients (^#^) serial dilutions of DNA were performed, with 0.5 logs step dilutions. The quantification of malignant cells in each cell fraction was calculated according to the single-hit Poisson model, and expressed as tumor cell frequency (1:x). The logarithmic decrease (lower part of cells) was calculated with respect to the foregoing unpurged sample indicated with "0". When the frequency of tumor contamination was below the detection threshold of the system, PCR were scored as negative (clear-shaded cells). The pooled data represent frequency determinations, according to the statistical method by Taswell.

**N.D**. = not done

**Table 3:** Statistical comparison of tumor load between different cell populations

	**ESTIMATED DECREASE (RATIO)**	**95% CONFIDENCE**	**INTERVAL**	**P VALUE**
**CD34^+ ^fresh cells versus CD34^+ ^cells after culture**	6.86	1.75	27.17	5.78 × 10^-3^
**CD34^+ ^fresh cells versus ΔNGFR^+ ^selected cells**	16.98	5.54	52.04	8.29 × 10^-7^
**CD34^+ ^after culture versus ΔNGFR^+ ^selected cells**	2.46	0.43	14.15	3.12 × 10^-1^

The amount of purging was evaluated in different coupled cell populations, and expressed as ratio of contaminant tumor cells (number of tumor cells pre purging/number of tumor cells after purging).

### Multiple myeloma cells: transduction and division pattern

**Figure 2:**
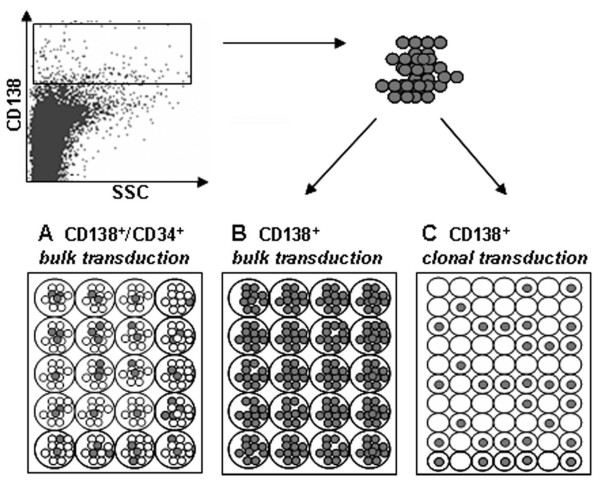
**Transduction pattern of myeloma plasma cells**. CD138+ selected myeloma bone marrow plasma cells were transduced with the ΔNGFR vector in a bulk, and in a clonal culture (B and C). Part of the cells was mixed before transduction with CD34+ cells of the same patient (A). Culture conditions were the same as in the CD34+ cell transductions.

To assess the transduction rate of myeloma cells, we infected BM CD34^+ ^cells of a MM patient, after adding back to the cell population CD138^+ ^myeloma plasma cells sorted from the same patient (ratio 1:1). By this way we reproduced our experimental condition setting, which includes a mixed population of healthy CD34^+ ^cells and myeloma cells. In addition, in a parallel experiment, we transduced CD138^+ ^sorted cells at 1 × 10^6^/well, to assess the transduction rate on a consistent number of cells, and at 1 cell/well, to study the division rate of tumor cells during transduction and therefore the possibility of the single cell to be infected by retroviral vectors (Figure 2).

Clonal transduction was performed at the same concentration of cytokines and virus as in the standard cell transduction, and the division pattern scored during the culture by light microscope (n = 89 single-cell wells). At the end of procedure we stained each well with Trypan-blue dye, to assess the viability of the cells. Whereas transduction rate of CD34^+ ^cells was 21.4% in the mixed cell population, no CD138^+ ^cell was transduced, neither in this cell population, nor in the CD138^+ ^selected cells. The rate of CD138^+ ^clonal cell division during viral exposition was 3.7%, but all cells died before the end of procedure, as assessed by Trypan blue labelling.

Previous studies report that LTMC can sustain myeloma cells [20]. To exclude the presence of myeloma "long-living" contaminants, or possible myeloma precursors in the transduced and selected cell fraction, we seeded CD34^+^/ΔLNGFR^+ ^cells of patient MM18 in a LTMC assay, adding IL6 to the culture to favour the maintenance of the plasma cells in culture. After 3 weeks, the cells were placed in a CFU-C assay, picked at the end of the culture, and analyzed for specific myeloma rearrangements. 100% of the colonies (42 out of 42) tested resulted negative for the patient-specific rearrangement (data not shown).

## Discussion

Available clinical evidences suggest that CD34^+ ^selection reduces myeloma contamination in PBSC (Peripheral Blood Stem Cell) collections [5,6,24,25], however, it does not eliminate it, and it does not improve disease-free or overall survival of transplanted patients [8,9,26]. We sought to improve the molecular purging of leukaphereses for autografting by exploiting the *in-vitro *biological behaviour of myeloma cancer cells. The results achieved with our protocol strongly suggest that optimised short-culture conditions provide a major contribution to purging: rearrangement-undetectable CD34^+ ^cells were generated in 57% (4 out of 7) samples. To further improve the purging results, we exposed CD34-selected cells to a retroviral transduction with a selectable membrane-associated marker. This innovative combined procedure exploits the gene transfer technique to enrich for high repopulating-capacity CD34^+ ^cells, and, at the same time, to eliminate myeloma contaminants. Moreover, an advantage of this approach is that it foresees the outcome of a possible future gene therapy addressed to the CD34^+ ^cells and their progeny.

The transduction and selection results indicate that the procedure is clinically feasible, with a mean amount of 22.3 ± 15% final transduced and selected CD34^+ ^cells, with respect to the initial CD34^+ ^cell number. We have previously demonstrated that ΔLNGFR^+ ^selected CD34^+ ^cells are capable of developing a multilineage reconstitution profile in a relevant animal model [18]. Previous reports have already assessed *ex-vivo *culture for purging of tumor cells in myeloma and lymphoma [27,28], but the long culture period (7–21 days) render these cells unsuitable for reinfusion.

We could not correlate our purging efficiency with cell expansion, neither with transduction efficiency, nor with cell recovery. The only parameter we could link with a poor purging outcome was the ΔNGFR purity of selection. Although it occurred in a single sample, a ΔNGFR purity of <93% correlated with the persistency of tumor contamination after culture and selection of transduced cells (see Table 1 and 2).

ΔNGFR^+ ^selection appeared to improve the tumor load removal, with respect to the simple culture-based purging. However, we did not observe a complete elimination of residual myeloma contaminants. DNA deriving from dead cells, still present at the end of the procedure, or actual tumor contaminants could explain the incomplete purging. Our experiments show a very low probability for plasma cells to be transduced in these culture conditions. Rather, the involvement of myeloma plasma cells in ΔLNGFR selection could occur because of their inherent characteristics, leading them to adhere via surface Ig to beads-coated antibodies. Since we did not reach a complete purging in ΔLNGFR^+ ^selected cells, we performed a clonogenic analysis on a ΔLNGFR^+ ^contaminated cell fraction which suggested that the myeloma contaminants present in the final cell fraction do not retain clonogenic capacity.

We previously demonstrated the multilineage reconstitution potential of MPB CD34^+ ^cells treated with the same culture and transduction protocol in SCID-hu animal models [18]. This particular animal model allows the differentiation of lymphocytes in a suitable microenvironment. We showed a maintained lymphoid reconstitution potential, with differentiation of mature B and T cells after CD34^+ ^cell transplant. We assume that a good and early lymphoid cell reconstitution is necessary in myeloma patients undergoing autologous transplants to protect against post-transplant infections [13], and probably against early tumor relapse. Nevertheless, we acknowledge the need for a more stringent assay of myeloid repopulating capacity, in order to move the technique into clinical application.

Recently, several clinical leukemia events reported by Hacein-Bey-Abina et al. in severe combined immunodeficiency-X linked (SCID-X) patients treated with γ-chain transduced CD34^+ ^cells [29] raised questions about the general safety of retrovirus-based gene therapy. The issue is still under intensive investigation with extensive analyses performed on transduced cells [30]. So far, no other group has reported similar adverse events, despite a high number of clinical trials performed in more than 10 years of gene therapy and gene marking studies [31]. Still, a justified caution in evaluating risks and benefits of a gene therapy clinical procedure is now mandatory.

Among the performed sequential purging steps, the CD34 selection was the least efficient: we could also consider applying the culture protocol directly to the whole leukapheresis. Provided that we reach experimental evidences of a similar purging, this setting would simplify the cell manipulation process and render the procedure more suitable for a clinical application.

## Conclusion

We conclude that the culture purging approach we have devised could be a feasible and efficient procedure for MM patients undergoing autologous transplantation. There is also a tendency of myeloma contaminants to further decrease after transduction and selection of CD34^+ ^cells, as compared with the simple culture. An advantage of the transduction protocol is a potentially myeloma-free population of marked cells, detectable over time. Gene marking of autografted cells has been shown to be a valuable tool to trace long-term reconstitution of the host [32], and the origin of disease relapse [33]. A pilot study for MM patients eligible to high dose chemotherapy and autologous transplant would clarify the effectiveness of the purging transplant approach described in this work. The safe reconstitution capacity of CD34^+ ^transduced cells could also allow a future gene therapy trial directed to hematopoietic CD34^+ ^cells.

## Abbreviations

ΔNGFR: truncated form of the human Nerve Growth Factor Receptor

BM: Bone Marrow

CFU-C: Colony Forming Units-Cells

CI: Confidence Interval

GvHD: Graft versus Host Disease

IgH: Immunoglobulin Heavy chain

IL: Interleukin

LTMC: Long-Term Marrow Cultures

MC: Method of likelihood maximization and χ^2 ^minimization

MM: Multiple Myeloma

MoMLV: Moloney Murine Leukemia Virus

MPB: Mobilized Peripheral Blood

PBSC: Peripheral Blood Stem Cell

SCID-hu: Severe Combined Immunodeficiency-humanized

SCID-X: Severe Combined Immunodeficiency-X linked

SN: Viral Supernatant

## Declaration of competing interests

The author(s) declare that they have no competing interests.

## Authors' contributions

SD performed experiments, analyses and wrote the paper.

SS, RSC, MC, FF, JD performed cell culture experiments and analyses.

CV and MM created the patient specific sequences, and analysed the molecular purging data

SV performed statistical analyses.

EP, GC, JP, CB, MGR and AA participated in the study design.

MB conceived the study, coordinated the work, and helped in writing the paper.

All authors read and approved the final manuscript.
